# Preliminary Investigation of *Astragalus arpilobus* subsp. *hauarensis*: LC-MS/MS Chemical Profiling, In Vitro Evaluation of Antioxidant, Anti-Inflammatory Properties, Cytotoxicity, and In Silico Analysis against COX-2

**DOI:** 10.3390/antiox13060654

**Published:** 2024-05-27

**Authors:** Sabrina Lekmine, Ouided Benslama, Kenza Kadi, Abir Brik, Ouidad Djeffali, Manar Ounissi, Meriem Slimani, Mohammad Shamsul Ola, Omayma A. Eldahshan, Antonio Ignacio Martín-García, Ahmad Ali

**Affiliations:** 1 Biotechnology, Water, Environment and Health Laboratory, Abbes Laghrour University, Khenchela 40000, Algeria; 2 Department of Molecular and Cellular Biology, Faculty of Natural and Life Sciences, Abbes Laghrour University, Khenchela 40000, Algeria; 3 Laboratory of Natural Substances, Biomolecules, and Biotechnological Applications, Department of Natural and Life Sciences, Larbi Ben M’Hidi University, Oum El Bouaghi 04000, Algeria; 4 Department of Biochemistry, College of Science, King Saud University, Riyadh 11451, Saudi Arabia; 5 Department of Pharmacognosy, Faculty of Pharmacy, Ain Shams University, Cairo 11566, Egypt; 6 Center for Drug Discovery Research and Development, Ain Shams University, Cairo 11566, Egypt; 7 Estación Experimental del Zaidín (CSIC), Profesor Albareda 1, 18008 Granada, Spain; 8 Department of Life Sciences, University of Mumbai, Vidyanagari, Mumbai 400098, India

**Keywords:** *Astragalus arpilobus* subsp. *hauarensis*, LC–ESI–MS/MS, antioxidant, anti-inflammatory, cytotoxicity, molecular docking, MD simulation

## Abstract

The search results offer comprehensive insights into the phenolic compounds, antioxidant, anti-inflammatory, cytotoxic effects, LC-MS/MS analysis, molecular docking, and MD simulation of the identified phenolic compounds in the *Astragalus arpilobus* subsp. *hauarensis* extract (AAH). The analysis revealed substantial levels of total phenolic content (TPC), with a measured value of 191 ± 0.03 mg GAE/g DM. This high TPC was primarily attributed to two key phenolic compounds: total flavonoid content (TFC) and total tannin content (TTC), quantified at 80.82 ± 0.02 mg QE/g DM and 51.91 ± 0.01 mg CE/g DM, respectively. LC-MS/MS analysis identified 28 phenolic compounds, with gallic acid, protocatechuic acid, catechin, and others. In the DPPH scavenging assay, the IC_50_ value for the extract was determined to be 19.44 ± 0.04 μg/mL, comparable to standard antioxidants like BHA, BHT, ascorbic acid, and α-tocopherol. Regarding anti-inflammatory activity, the extract demonstrated a notably lower IC_50_ value compared to both diclofenac and ketoprofen, with values of 35.73 µg/mL, 63.78 µg/mL, and 164.79 µg/mL, respectively. Cytotoxicity analysis revealed significant cytotoxicity of the *A. arpilobus* extract, with an LC_50_ value of 28.84 µg/mL, which exceeded that of potassium dichromate (15.73 µg/mL), indicating its potential as a safer alternative for various applications. Molecular docking studies have highlighted chrysin as a promising COX-2 inhibitor, with favorable binding energies and interactions. Molecular dynamic simulations further support chrysin’s potential, showing stable interactions with COX-2, comparable to the reference ligand S58. Overall, the study underscores the pharmacological potential of *A. arpilobus* extract, particularly chrysin, as a source of bioactive compounds with antioxidant and anti-inflammatory properties. Further research is warranted to elucidate the therapeutic mechanisms and clinical implications of these natural compounds.

## 1. Introduction

Oxidative stress, a condition characterized by an imbalance between oxidants and antioxidants in favor of oxidants, can have significant impacts on the body. This imbalance leads to disruptions in redox signaling, molecular damage, and the potential destruction of cells [1].

Oxidative stress can be further categorized into oxidative stress, which involves low to mild levels of oxidants regulating various pathways, and oxidative distress, which describes a condition leading to damage in biological components like proteins, membranes, and DNA [2].

The consequences of oxidative stress are far-reaching and can contribute to inflammation within the body. Reactive oxygen species (ROS) generated during oxidative stress can trigger inflammation, impacting processes like immune cell activation, ischemia, infection, cancer, excessive exercise, mental stress, and aging [3].

In the realm of plant-based medicine, it is commonly observed that if a plant exhibits anti-inflammatory effects, it logically follows that it also possesses antioxidant properties [3]. This correlation stems from the interconnected nature of inflammation and oxidative stress within the body. Inflammation is often triggered by the body’s response to harmful stimuli, leading to the production of reactive oxygen species (ROS) and free radicals. These molecules can cause oxidative damage to cells and tissues, contributing to various health issues. By exerting anti-inflammatory effects, a plant helps reduce inflammation and subsequently lowers the production of ROS, thereby exhibiting antioxidant activity [4]. This dual action showcases the holistic approach of plants in combating oxidative stress and inflammation, highlighting their potential as natural remedies for promoting overall health and well-being [4,5].

Many medicinal plants contain compounds with anti-inflammatory properties such as flavonoids, polyphenols, and terpenes [6]. These compounds can inhibit inflammatory signaling pathways and reduce the production of inflammatory mediators [7]. As a result, extracts with strong anti-inflammatory activity like casapon mitigate inflammatory responses in the body, which may contribute to reducing cytotoxicity associated with chronic inflammation [7]. Some medicinal plant extracts may also have cytotoxic properties, meaning that they can be toxic to cells [8,9,10,11]. In the context of extracts used to treat cancer, for example, selective cytotoxic activity can be beneficial by specifically targeting cancer cells while preserving normal cells [12]. However, this cytotoxicity needs to be selective and not excessively damage healthy tissues. In summary, medicinal plant extracts can synergistically provide antioxidant, anti-inflammatory, and cytotoxic effects. These activities may be interconnected and mutually reinforce each other to promote health and well-being, but it is also essential to understand the specific mechanisms of action of each medicinal plant for safe and effective use.

One of the primary botanical groups, the Fabaceae, is home to the majority of plants found in arid environments. This plant family is also frequently employed in traditional African medicine [12]. With over 2500–3000 annual and perennial species spread throughout all continents, mostly in the Northern Hemisphere, Western North America, South America, Central Asia, and tropical East Africa, *Astragalus* is arguably the largest and most common genus of vascular plants on Earth. *Astragalus* grows throughout North Africa and the Mediterranean in many different varieties; 15 species are reported in Algeria’s Sahara [13].

Three major classes of physiologically active chemicals found in several *Astragalus* plants have been thoroughly examined: polysaccharides, flavonoids, and saponins [14,15,16,17]. In North African medicine, *Astragalus arpilobus* subsp. *hauarensis* is traditionally used to cure snakebites. *A. arpilobus* is referred to as “Dlilia” locally and is widely distributed in Algeria’s deserts [18].

To our knowledge, the chemical composition and biological benefits of *A. arpilobus* extract (AAH) have not been reported. The present experimental study was conducted to investigate the anti-inflammatory and antioxidant effects of the *A. arpilobus* plant while confirming its safety through testing the plant extract against two types of crustacean cells, *Artemia salina*, to assess its cytotoxic effects. Additionally, the study aimed to quantify and identify the chemical compounds present in the tested plant using LC-MS-MS analysis.

Considering the pivotal role of inflammation in various diseases, the cyclooxygenase-2 (COX-2) enzyme has emerged as a promising target for anti-inflammatory interventions [19]. COX-2 is an inducible enzyme that catalyzes the synthesis of prostaglandins, which are lipid mediators involved in inflammation, pain, and fever responses [20]. Inhibiting COX-2 activity has been a central strategy in the development of nonsteroidal anti-inflammatory drugs (NSAIDs), which are widely used for the treatment of inflammatory conditions such as arthritis and other inflammatory disorders [21]. Therefore, the investigation of *A. arpilobus* extract’s potential inhibition of COX-2 activity holds significant therapeutic relevance and merits exploration for the development of novel anti-inflammatory agents.

In line with this rationale, molecular docking studies were conducted to elucidate potential interactions between select phenolic compounds from the *A. arpilobus* extract and the COX-2 enzyme. By integrating molecular docking analyses into our investigation, we aimed to uncover the structural basis underlying the extract’s pharmacological activities. This approach not only enhances our comprehension of the extract’s anti-inflammatory potential, but also lays the groundwork for the rational design of future therapeutic interventions targeting inflammatory pathways.

## 2. Materials and Methods

### 2.1. Plant Material and Extraction

Aerial parts of *A. arpilobus* were collected from the Wilaya El Oued in the northeastern Algerian Sahara. Upon harvesting, the plant material underwent meticulous processing. It was first air-dried and then finely pulverized into a powder. To obtain hydro-alcoholic extracts with a concentration of 70% ethanol, 20 g of plant powder was utilized. Subsequently, the powder was mixed with ethanol and allowed to dissolve at room temperature for a full day. After thorough dissolution, the mixture underwent filtration, and the filtrate was concentrated at 36 °C using a rotavapor (Hahnvapor, Hahnshin Scientific Co., Ltd., Seoul, Republic of Korea). The residue remaining after the hydro-alcoholic extraction was carefully collected and stored at 4 °C for future use.

### 2.2. Total Bioactive Compounds

The method used to quantify polyphenols in the extract followed the protocol detailed by Singleton and Rossi (1965). This process involved several successive steps. First, a sample was prepared by accurately weighing a portion of the extract (prepared with 4 mg of extract and 1 mL of 70% ethanol). Next, a solution of the Folin–Ciocalteu reagent was prepared according to the specific instructions of the method. Then, 200 µL of the sample was mixed with 1 mL of Folin–Ciocalteu solution and incubated in darkness for 4 min. After this incubation period, 800 µL of alkaline solution (sodium carbonate solution at 70 mg/mL) was added to the mixture, followed by another incubation phase (2 h at room temperature). Finally, the absorbance of the reaction mixture was measured at a wavelength of 765 nm using a spectrophotometer, and the concentration of polyphenols was determined by referring to a calibration curve previously established from standard solutions of polyphenols. The results were expressed as milligrams of gallic acid equivalents per gram of dry matter (mg GAE/g DM) [22].

The AAH extract was analyzed to assess its flavonoid concentration using spectrophotometry, following the aluminum chloride (AlCl_3_) protocol described by Ayoola et al., (2008) with specific adjustments. Initially, the extracts were prepared in methanol, and an aluminum chloride solution was then added to the extracts in test tubes (500 µL of the sample with 100 µL of AlCl_3_ solution). After adding 100 µL of potassium acetate solution to stabilize the reaction, the mixture was incubated at room temperature in the dark for 40 min. Subsequently, the solution’s absorbance was measured at a wavelength of 415 nm using a spectrophotometer. A calibration curve was established using standard solutions of flavonoids with known concentrations Quercetin was used as the reference compound, and the results were expressed as milligrams of quercetin equivalents per gram of dry matter (mg QE/g DM) [23].

The tannin content was evaluated according to the protocol outlined by Oyedmi and Afolayan (2011). This protocol entailed mixing 0.5 mL of extract solution with 3 mL of vanillin solution, followed by the addition of 1.5 mL of 37% hydrochloric acid (HCl). After agitation and a 15-min incubation period at room temperature, absorbances were measured at 500 nm. Additionally, a control sample was prepared by combining 0.5 mL of extract with 3 mL of a 70% ethanol and 30% water mixture, along with 1.5 mL of HCl. Subsequently, a calibration curve was constructed using catechin as a reference under the same conditions. The results were expressed as catechin equivalent per gram of dry matter (mg CE/g DM) [24].

### 2.3. Equipment and Chromatographic Parameters

For the LC-MS/MS analyses, samples containing 50 mg were transferred into Eppendorf tubes containing 2 mL of ethanol. The resulting solution was thoroughly stirred before undergoing extraction using hexane. Following extraction, the mixture underwent centrifugation and stirring at 9000 rpm for 10 min. Subsequently, a 100 µL sample was extracted from the methanol phases in the resulting solution and diluted to 900 µL with a ratio of 450 water to 450 methanol. Finally, the resulting sample underwent filtration and was analyzed by LC/MS-MS with a 5.0 µL injection volume, a flow rate of 0.400 mL/min, a method time of 30 min, and a temperature of 40 °C.

### 2.4. Antioxidant Activity

The DPPH test protocol, as indicated by Sanchez et al. (1998) [25], begins by dissolving 2.5 mg of DPPH in 100 mL of methanol to achieve an absorbance of (0.98 ± 0.02) at 517 nm. Subsequently, samples of extracts or standards (ascorbic acid, quercetin, and BHA) of various concentrations are mixed with 100 μL of this DPPH solution. The mixture is then incubated in the dark at room temperature for 30 min, followed by the measurement of absorbance at 517 nm. A decrease in the absorbance of the DPPH solution indicates antioxidant activity, while unchanged absorbance suggests the absence of antioxidant activity in the sample. The results were then compared to control solutions to evaluate the antioxidant efficacy of the tested sample. The percentage of DPPH radical scavenging activity of each extract is then calculated as follows:

% of DPPH radical scavenging activity = ((Ac − (At − Ae))/Ac) × 100%.

where:

Ac: Absorbance of the control (this is the absorbance of the blank containing only DPPH).
At: Absorbance of the test (this is the absorbance of the DPPH solution containing the extract).
Ae: Absorbance of the blank (this is the absorbance of the extract solution without DPPH).
The IC_50_ value, which represents the concentration of the sample required to scavenge 50% of the DPPH radicals, can be determined from the regression line where the inhibition ratio is 50%.

### 2.5. Anti Inflammatory Assay

The in vitro anti-inflammatory activity was evaluated using the protein denaturation method with bovine serum albumin (BSA), following the procedure outlined by Karthik et al. (2013) [26]. In this method, 0.5 mL of various concentrations of the extract or reference compounds (ketoprofen and diclofenac) were mixed with 0.5 mL of BSA (0.2% *w*/*v*) in Tris-HCl buffer (pH 6.8). The sealed tubes were then incubated in an oven at 37 °C for 15 min, followed by heating in a water bath at 70 °C for 5 min to induce protein denaturation. The absorbance of turbidity was measured at 660 nm using a spectrophotometer. The percentage inhibition of protein denaturation was calculated using a standard equation, with each experiment conducted in triplicate to ensure reliability. The IC_50_ was then determined and the results were expressed as means ± SD of three parallel measurements and compared with the reference compounds.

% I= [[Ac − (As − Aw)]/Ac] × 100.

As: Absorbance of samples: 0.5 mL extract + 0.5 mL BSA.
Aw: Absorbance of white: 0.5 mL extract + 0.5 mL Tris-HCL (pH: 6.8).
Ac: Absorbance of control: 0.5 H_2_O + 0.5 mL BSA (The control represents 100% of proteins denaturation).

### 2.6. Cytotoxic Effect by Brine Shrimp Lethality Test (BST)

The cytotoxic activity of the plant tested was assessed using the shrimp assay method [27]. Shrimp larvae hatching was initiated by placing about 100 mg of shrimp eggs into seawater at ambient temperature. A plastic container was used, divided into two compartments by a polyethylene glycol wall with a few holes. One compartment was covered with aluminum foil to create darkness, while the other was exposed to light. After hatching, the mature shrimp nauplii were attracted to and migrated toward the lighted compartment, where they were then collected for the cytotoxic bioassay.

For the assay, the extract was prepared in seawater at various concentrations (4, 2, 1, 0.5, 0.25, 0.125, and 0.0625 mg/mL). Each vial received 4 milliliters of seawater to reach a final volume of 5 mL, and then ten nauplii were added to each vial. A positive control using potassium permanganate was prepared in the same way, but without the extract. A negative control consisted of 0.1 mL of DMSO with ten nauplii and 4.9 mL of seawater.

All experiments were incubated for 24 h under light. The number of surviving nauplii was counted, and the mortality percentage for each concentration was determined. The median lethal concentration (LC50) was calculated; this value represents the concentration at which half of the nauplii die. Mortality percentage was evaluated using the formula:

$$Themortality\left(\%\right)=(Noofdeadnauplii/Totalnumber)\times 100$$

The lethality of the extract on the brine shrimp was classified as follows [28]:

LC50 ≥ 1000 µg·mL^–1^ was “nontoxic”;
LC50 = 500–1000 µg·mL^–1^ was “weakly toxic”;
LC50 = 100–500 µg·mL^–1^ was “moderately toxic”;
LC50 ≤ 100 µg·mL^–1^ was “strongly toxic [7,29].

### 2.7. Virtual Screening

#### 2.7.1. Retrieval and Preparation of Protein Structure

The 3-dimensional structure of cyclooxygenase-2 (COX-2) in complex with the inhibitor SC-558 (PDB ID: 1CX2), resolved at a 3.00 Å resolution, was obtained from the RCSB Protein Data Bank “www.rcsb.org (access on 8 April 2024)” in PDB format. To prepare the protein structure, various protein preparation modules within Auto Dock Vina Tools [30] and UCSF Chimera version 1.15 [31] were utilized. Initially, the NAG ligand, water molecules, and heteroatoms were eliminated, and any missing hydrogen atoms were added to the structure. Subsequently, Kollman charges were assigned to the structure, and the optimization and minimization of the structure were performed using the Amber force field ff14SB. The processed structure was finally saved in the Mol2 format.

#### 2.7.2. Phytochemicals Preparation

In addition to the co-crystallized ligand SC-558, the 3D conformations of the 38 identified phytocompounds were obtained from the PubChem database in SDF format. The Merck Molecular Force Field 94 (MMFF94), integrated into the Chimera package, was employed to optimize each molecule, and polar hydrogens and Gasteiger charges were subsequently added. Following optimization, the structures of the molecules were converted into the Mol2 format.

#### 2.7.3. Molecular Docking Protocol

Initially, the co-crystallized inhibitor SC-558 was docked using site-specific molecular docking to explore its interactions with COX-2 alongside the prepared 38 phytochemicals from A. arpilobus. The AutoDock Vina program facilitated the docking process, preceded by the generation of a ‘conf.txt’ file to specify the grid box and other parameters. A 3D grid box measuring 33 Å × 25 Å × 27 Å, centered at coordinates X22.63, Y19.89, and Z16.21, was constructed around the coordinates of the co-crystallized ligand. This grid box en-compassed the binding site’s static conformation, and the docking simulations comprised a total of 50 genetic runs with a default exhaustiveness value of 8. Subsequently, the top-ranking molecules were selected based on their binding affinity and number of hydrogen bonds. The re-docking involved re-docking the co-crystallized inhibitor SC-558 into the active site of COX-2 using AutoDock Vina. The grid box dimensions and docking parameters remained consistent with the initial docking protocol to ensure a direct comparison between the re-docking results and the crystallographic binding mode of SC-558. The re-docked poses were then analyzed based on their binding affinity, RMSD values, and consistency with the crystallographic conformation of SC-558 in complex with COX-2. This evaluation provided valuable insights into the reliability and predictive capability of the molecular docking protocol utilized in this study. To visualize and annotate the molecular interactions both in 3D and 2D modes, Discovery Studio was employed. Additionally, the pharmacophores of the top molecules were generated using the software MOE 2015.10 to conduct a structure-activity relationship (SAR) analysis.

#### 2.7.4. Molecular Dynamics Simulation

MD simulations were performed utilizing the NAMD 2.14 package [23] to evaluate the stability of docking complexes comprising the top-ranked bioactive ligand, chrysin, alongside the co-crystallized inhibitor S58 with the COX-2 enzyme. Topology data for the ligands were automatically generated through the CHARMM-GUI server employing the charmm36 force field [24]. Solvation and neutralization of the systems were accomplished using the TIP3P water model, supplemented with Na^+^ and Cl^−^ ions. The stimulation protocol commenced with 5000 steps of energy minimization, followed by an equilibration phase under constant volume and temperature (NVT). Subsequently, a production phase of 100 ns at a time step of 2 fs was conducted under constant temperature and pressure of 300 K (NPT). The analysis of simulation trajectories encompassed the root mean square deviation (RMSD), root mean square fluctuations (RMSF), the radius of gyration (Rg), solvent accessible surface area (SASA), and hydrogen bond count. The g_mmpbsa tool was used to assess the binding free energies of the enzyme–ligand complexes [25]. The MMPBSA analysis was performed for the last 5 ns of the MD trajectory for each enzyme–ligand complex.

### 2.8. Statistical Analysis

The statistical analysis of the data was conducted utilizing GraphPad Prism Data Editor 6.0. Initially, one-way ANOVA was employed to analyze the data, followed by Dunnett’s test to identify significant differences between the test and control groups. The results were expressed as the mean ± standard error of the mean. A *p*-value of less than 0.05 was considered statistically significant, indicating meaningful differences between the experimental groups. This comprehensive analysis allowed for the accurate interpretation of the data and the identification of significant outcomes within the study.

## 3. Results

### 3.1. Total Bioactive Compounds, Antioxidant, Anti-Inflammatory, and Cytotoxic Activities

After extracting the plant, the obtained extract (4 g) was subjected to several biological activity assessments. The analysis revealed that the AAH extract exhibited a substantial total phenolic content (TPC) of 191 ± 0.03 mg GAE/g DM (Table 1). This noteworthy TPC predominantly arises from two primary phenolic compounds: the total flavonoid content (TFC) and total tannin content (TTC). Specifically, the TFC was quantified at 80.82 ± 0.02 mg QE/g DM, while the TTC was measured at 51.91 ± 0.01 mg CE/g DM.

On the other hand, the IC_50_ values in scavenging DPPH radicals, as outlined in Table 1, serve as critical indicators of the antioxidant potential exhibited by both the plant extract and various standard compounds. It is important to note that a smaller IC_50_ value corresponds to higher antioxidant activity, representing the concentration required to neutralize 50% of the DPPH radicals present. In this study, the IC_50_ value for the AAH extract was determined to be 19.44 ± 1.1 µg/mL. Comparing the IC_50_ values between the AAH extract and the standard compounds elucidates their relative antioxidant efficacy. The plant extract demonstrated a favorable IC_50_ value closer to BHA, BHT, ascorbic acid, and α-tocopherol, indicating its potential as an effective antioxidant agent.

A significant difference was observed between the IC_50_ value of the plant and that of ascorbic acid. However, no significant difference was observed with the other standards.

The IC_50_ values for the anti-inflammatory activity of the AAH extract and the standard drugs diclofenac and ketoprofen were determined to be 35.73 ± 1.3 µg/mL, 63.78 ± 2.1 µg/mL, and 164.79 ± 2.3 µg/mL, respectively (Table 1). These values represent the concentration at which the compounds can inhibit inflammation by 50%. A significant difference was noted between the AAH extract and the standard drugs diclofenac and ketoprofen. This suggests that the AAH extract possesses stronger anti-inflammatory properties relative to the standard drugs.

The mortality results are also presented in Table 1. No mortality was observed in the control group treated with DMSO. However, the AAH extract demonstrated significant cytotoxicity with an LC_50_ value of 28.84 ± 2.1 µg/mL, which exceeded that of potassium dichromate, having an LC_50_ of 15.73 µg/mL. This suggests that the AAH extract has a lower cytotoxic effect compared to potassium dichromate, indicating its potential as a safer alternative for various applications.

### 3.2. LC-MS/MS Analysis

The results of our qualitative analysis revealed the presence of key phenolic compounds within the AAH extract, shedding light on the chemical composition of this botanical extract. Table 2 provides a comprehensive overview of the major phenolic constituents detected in the AAH extract.

The phytochemical composition of the plant extract, as assessed via LC-MS, revealed a diverse array of phenolic compounds and flavonoids. These phenolic acids are well-recognized for their robust antioxidant and anti-inflammatory properties, indicating the potential health-promoting effects of the plant extract.

The flavonoid class was characterized by distinct subgroups, with flavan-3-ols represented by catechins, which were also identified in the analyzed plant extract. These compounds contribute to the diverse array of phytochemicals present, each with unique properties and potential health benefits. Additionally, various flavones including baicalein aglycone and scutellarin glucuronide were identified as well as flavonols such as morin, kaempferol, quercetin, and fisetin. The flavanones hesperetin and naringenin were also identified, along with the isoflavone biochanin A. Interestingly, glycosylated forms of flavonoids such as quercetin-deoxyhexosyl hexoside, hesperetin-deoxyhexosyl hexoside, quercetin pentoside, and kaempferol hexoside were also present in the extract. This diverse phytochemical profile highlights the rich source of bioactive compounds in the analyzed plant material, with potential applications in various areas of health and nutrition.

### 3.3. Molecular Docking

**Figure 1:**
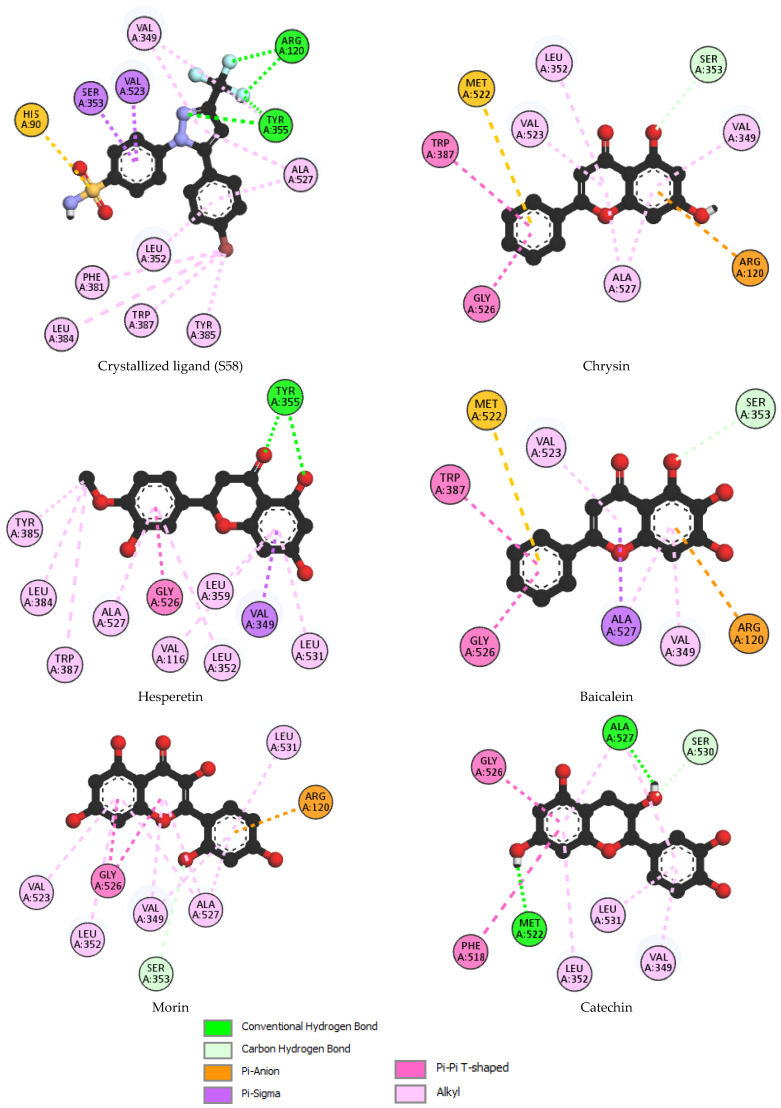
2D interaction modes of S58 and the best docked phytocompounds with COX-2 (PDB:1CX).

**Figure 2:**
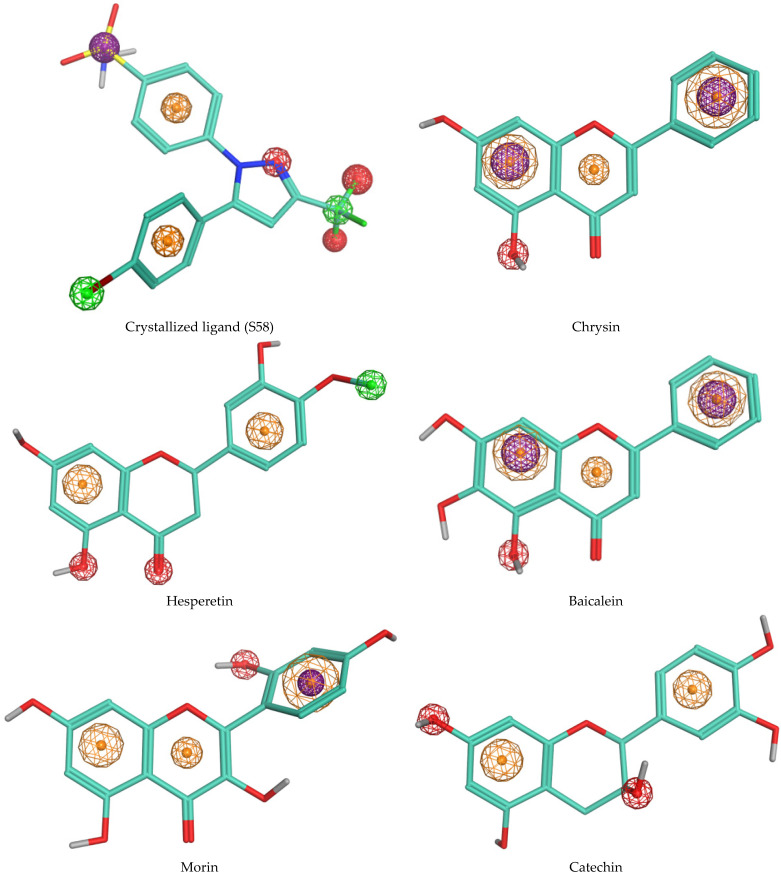
Pharmacophore modeling of S58 and the best docked phytocompounds. The red contour represents acceptor/donor H-bond (Acc/Don), the green contour represents hydrophobic region, the orange contour represents aromatic/hydrophobic regions, and purple contour represents Pi-charge electrostatic forming regions.

In this study, we employed molecular docking to investigate the interactions between phenolic compounds identified in the AAH extract and cyclooxygenase-2 (COX-2). Prior to discussing the docking results, it is essential to note that a validation step was conducted to assess the reliability of our docking protocol. The re-docking procedure, aimed at evaluating the accuracy of our docking simulations, yielded a root mean square deviation (RMSD) value of 0.651. This RMSD value indicates the degree of deviation between the re-docked poses of the co-crystallized inhibitor SC-558 and its crystallographic conformation in complex with COX-2. With this validation in place, we proceeded to analyze the docking results to elucidate the binding modes and interactions of the identified phenolic compounds with COX-2. Among the molecules identified in the extract, five compounds exhibited the most favorable binding scores with COX-2, as depicted in Table 3 and Figure 1. Furthermore, to provide visual insights into the specific molecular features contributing to the observed binding modes, the pharmacophore modeling of S58 and the best-docked phytocompounds is presented in Figure 2.

### 3.4. Molecular Dynamic Simulations

**Figure 3:**
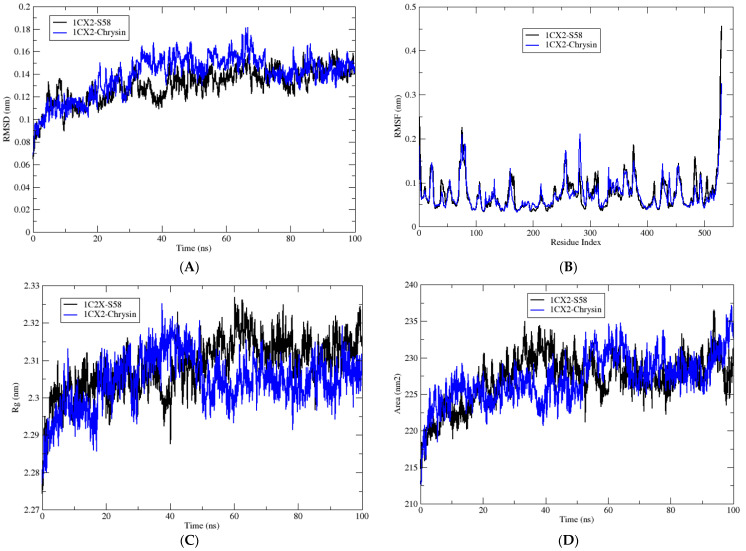

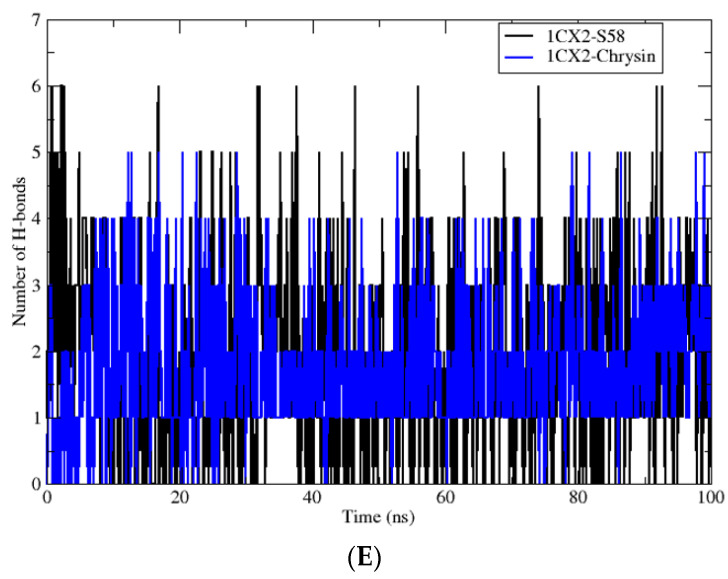
(**A**) RMSD of the complexes 1CX2-S58 and 1CX2-Chrysin observed during 100 ns MD simulation. (**B**) RMSF of the complexes 1CX2-S58 and 1CX2-Chrysin during 100 ns MD simulation. (**C**) The radius of gyration of the complexes 1CX2-S58 and 1CX2-Chrysin during 100 ns MD simulation. (**D**) SASA of the complexes 1CX2-S58 and 1CX2-Chrysin observed during 100 ns MD simulation. (**E**) Number of H-bonds of the two complexes 1CX2-S58 and 1CX2-Chrysin during 100 ns MD simulation.

In this part of the study, the biological system formed by the target enzyme in complex with the inhibitor S58 was used as a reference for comparison with the complexes formed by the same target enzyme with the phenolic compound producing the best result in the molecular docking analysis, chrysin. The results are summarized in Table 4 and Table 5 and Figure 3.

## 4. Discussion

Our study represents the first exploration in the literature focusing specifically on the phenolic compounds of *A. arpilobus.* While past research has examined the phenolic compounds of various *Astragalus* species, none have specifically addressed the phenolic compounds of *A. arpilobus* until now. This underscores the novelty and importance of our findings in expanding the understanding of the phytochemical composition and potential health benefits of *A. arpilobus.*

Numerous phenolic compounds identified in our plant through LC-MS/MS analysis are also present in various species of *Astragalus* such as gallic acid, a prominent phenolic compound that has undergone extensive investigation for its antioxidant and anti-inflammatory properties [32]. Additionally, the phenolic compounds mentioned earlier were identified in the methanol extract of *A. schizopterus* [33]. Likewise, significant phenolic compounds like kaempferol, quercetin, and rutin were detected in various *Astragalus* species using two distinct HPLC [34,35]. Similar phenolic compounds were isolated and purified from *A. taipaishanensis*, with their structures elucidated utilizing techniques such as ESI-MS, HR-ESI-MS, 1D-NMR, and 2D-NMR. Among these compounds are quercetin, kaempferol, p-hydroxybenzoic acid, and vanillic acid [36], all of which were successfully detected in our current study.

In their study, Lekmine et al. [5] noted both similarities and differences in the presence of phenolic compounds compared to those identified in our current research. These differences in compound composition and quantity can be attributed to several factors. First, ecological conditions such as soil composition, altitude, and exposure to sunlight can significantly influence the production of secondary metabolites in plants [37,38]. Moreover, genetic differences between plant species or even within the same species can lead to variations in metabolite production [39]. Furthermore, environmental stressors such as pollution, drought, or herbivory can trigger plants to produce specific compounds as defense mechanisms, further contributing to the observed differences [40].

Finally, the geographical locations where the plant materials were collected can play a crucial role, as distinct regions may have unique microclimates and soil compositions, influencing the overall chemical profile of the plants [41]. Therefore, the divergence in compound composition between studies underscores the complexity of plant chemistry and highlights the importance of considering multiple factors when comparing phytochemical profiles across different studies. Based on the literature and the present results, we can consider the AAH extract an important source of phenolic compounds with potential for biomedical applications due to its secondary metabolism, which is dependent on local ecological conditions.

According to our results, the comparison showed that although the AAH extract exhibited slightly lower inhibition compared to BHA, BHT, ascorbic acid, and α-tocopherol, it still demonstrated considerable antioxidant efficacy compared to some other plant extracts and other antioxidant methods [42]. This suggests that the AAH extract possesses noteworthy potential as a natural antioxidant agent.

Numerous studies focusing on the same genus have consistently demonstrated potent antioxidant activity. For example, the ethanolic extract of *A. armatus* showcased significant antioxidant potential, as evidenced by its positive results across seven distinct antioxidant assays [5].

Similarly, *A. monspessulanus* exhibited moderate activity by using the DPPH test with an IC_50_ value of 13.57 ± 1.5 μg/mL, which closely aligns with our result of 19.44 ± 1.1 μg/mL [4].

Compared to our results, a slight difference in IC_50_ was observed in the study by Lekmine et al. (2020) on the butanol extract from flowers *of A. gombifirmis*, with an IC_50_ value of 16.43 ± 0.46 in the DPPH test [42].

Concerning the anti-inflammatory potential of the test plant, bovine serum albumin (BSA) is a globular protein that contains aromatic amino acids such as tryptophan, tyrosine, and phenylalanine [43]. These amino acids are responsible for the protein’s anti-inflammatory activity when it undergoes denaturation. The denaturation of BSA can be used as a model to evaluate the anti-inflammatory properties of various substances such as plant extracts [43]. The anti-inflammatory activity of BSA denaturation is measured by the inhibition of the denaturation process itself. When a substance with anti-inflammatory properties is present, it can prevent or slow down the denaturation of BSA, indicating its ability to reduce inflammation [44].

The anti-inflammatory potential of the AAH extract was evaluated. This process involves the disruption of electrostatic, hydrophobic, hydrogen, and disulfide bonds crucial for maintaining the 3-dimensional structure of proteins [45]. When proteins undergo denaturation, they often lose their biological functions, leading to the production of autoantigens that can trigger various autoimmune dysfunctions including rheumatic and inflammatory diseases. Consequently, compounds that can inhibit protein denaturation are highly regarded as effective treatments for arthritis and inflammation [46].

The results of in vitro anti-inflammatory activity revealed that the AAH extract exhibited remarkable efficacy in preserving the 3-dimensional structure of proteins, suggesting its potential as a therapeutic agent for combating inflammation. Our study represents the inaugural research in the literature dedicated to exploring the anti-inflammatory properties of *A. arpilobus*, filling a significant gap in the existing literature where information on this plant is lacking.

Several studies have investigated the anti-inflammatory activity of *Astragalus* species using the BSA denaturation method. *A. membranaceus* (Fisch.) Bunge has been shown to exhibit anti-inflammatory activity through the inhibition of pro-inflammatory mediator production [47]. A commercial extract of *A. membranaceus* has been found to attenuate inflammation and oxidative stress in intestinal cells by enhancing Nrf2 activation and HO-1 and NQO1 expression [47]. *A. polysaccharides* has been reported to have effects on the activation of B cells and macrophages, the promotion of humoral and immune responses, protection of blood vessels, and prevention of inflammation and cancer [48]. Triterpene derivatives from *Astragalus* have been reported to have anti-inflammatory effects [15].

A strong correlation was found between the anti-inflammatory activity, antioxidant, and phenolic compounds previously identified using the LC–ESI–MS technique [49]. Gallic acid, protocatechuic acid, chlorogenic acid, hydroxybenzaldehyde, vanillic acid, syringic acid, salicylic acid, *trans*-ferulic acid, sinapic acid, *p*-coumaric acid, and protocatechuic ethyl ester are phenolic acids known for their antioxidant and anti-inflammatory effects [50].

These compounds exhibit anti-inflammatory activity by modulating pro-inflammatory mediators like cyclooxygenase (COX) and transcriptional elements involved in antioxidant pathways such as nuclear factor-κB (NF-κB) and nuclear factor-erythroid factor 2-related factor 2 (Nrf-2) [50].

Additionally, flavonoids like catechin, scutellarin, quercetin, naringenin, hesperetin, kaempferol, baicalein, luteolin, biochanin A, quercetin-3-xyloside, hesperetin-deoxyhexosyl hexoside, quercetin pentoside, morin, kaempferol hexoside, fisetin, baicalein hexouronide, and chrysin also possess antioxidant and anti-inflammatory properties [51].

However, it is important to note that while these identified compounds may contribute significantly to the anti-inflammatory and antioxidant effects observed, there could be other bioactive molecules present in the extract that were not detected via LC-MS analysis. These undetected molecules such as fatty acids, carotenoids, and steroids may also play a significant role in the observed anti-inflammatory capacity of the extract [52].

Botanical extracts such as the AAH extract investigated in our study offer a complex mixture of phytochemicals including phenolic compounds that may exhibit synergistic or antagonistic effects on biological activities [53].

When comparing concentrations of extracts with pure polyphenols, it is crucial to recognize that the efficacy of an extract is not solely determined by the abundance of a particular molecule. Indeed, an extract may contain a high proportion of a specific reference molecule that contributes little to its overall antioxidant or anti-inflammatory activity. Conversely, other components within the extract may exert significant biological effects, even at lower concentrations [54].

This variability in activity underscores the importance of evaluating extracts holistically, considering the collective contribution of all phytochemical constituents to the observed biological effects. Moreover, the complexity of botanical extracts presents challenges in standardizing dosage and ensuring consistency across studies, further emphasizing the need for careful interpretation when comparing concentrations with pure polyphenols or reference molecules [55].

While our study focused on quantifying individual phenolic compounds within the AAH extract, future research could explore the interactions between these compounds and their collective impact on biological activity. Additionally, investigating the presence of nonphenolic bioactive molecules within the extract could provide further insights into its therapeutic potential.

The majority of plants exhibited anti-inflammatory and antioxidant effects, attributed to their diverse medicinal properties. While many plants offer these benefits, some also possess anticancer properties, often attributed to their capability to disrupt cell walls. In our research, we subjected our plant sample to testing against eukaryotic cells of *Artemia salina*. This experimentation aimed to ascertain the potential toxicity of our plant extract toward eukaryotic cells. Such investigations are crucial for determining the safety and efficacy of plant-derived compounds for potential therapeutic applications. The brine shrimp bioassay is used to assess the potential toxicity of bioactive compounds present in extracts that are typically toxic at high doses. This bioassay involves testing the lethality of a sample on brine shrimp (*Artemia salina*), a simple zoological organism. The concentration-dependent increase in brine shrimp mortality indicates the cytotoxic effects of plant extracts, which are reported to possess antimicrobial and antifungal properties [29].

The brine shrimp bioassay is a valuable tool for screening chemical compounds for biological activities. It is based on the premise that toxicology can be considered as pharmacology at higher doses, suggesting that if toxic compounds are found, lower nontoxic doses might exhibit useful pharmacological effects. This assay is widely used to detect a broad range of biological activities and chemical structures, making it a versatile method for the preliminary screening of bioactive substances in plant extracts [56].

Many researchers have found that extracts from different species of the *Astragalus* genus exhibit varying degrees of cytotoxicity against *Artemia salina*. These studies typically involve preparing extracts from different plant parts (such as roots, leaves, or seeds) using various extraction methods (such as solvent extraction or maceration) [57]. The cytotoxic activity of these extracts is then assessed by exposing *Artemia salina* nauplii to different concentrations of the extract and monitoring their mortality rate over a specific period. Results from these studies have demonstrated that certain extracts from the *Astragalus* genus possess significant cytotoxic activity against *Artemia salina*, indicated by a concentration-dependent increase in mortality rate [58]. The cytotoxic effects observed in these extracts may be attributed to the presence of bioactive compounds such as flavonoids, saponins, alkaloids, and phenolic compounds, which are known for their cytotoxic properties [59].

Numerous flavonoids have been investigated for their cytotoxic effects on cancer cells, showing promising potential as anticancer agents [47]. Among these molecules are quercetin, kaempferol, luteolin, apigenin, hesperetin, and naringenin [47]. These compounds were detected in our plant sample using LC-MS-MS analysis, suggesting that they may contribute to the observed anti-cytotoxic activity of the plant extract. The presence of such diverse flavonoids underscores the potential pharmacological significance of the plant and highlights its potential for therapeutic applications [60].

On the other hand, these flavonoids have been extensively studied for their ability to induce cytotoxicity in various cancer cell lines [61]. Quercetin, for example, has been shown to inhibit the proliferation of cancer cells and induce apoptosis [61]. Kaempferol exhibits similar effects, along with potential anti-metastatic properties [62].

Luteolin, apigenin, hesperetin, and naringenin are also flavonoids that have shown cytotoxic effects on cancer cells in various studies [63]. Their mechanisms of action may involve the induction of apoptosis, cell cycle arrest, inhibition of angiogenesis, and modulation of signaling pathways associated with cancer progression [63]. Overall, these flavonoids represent a diverse group of compounds with significant potential as anticancer agents. Further research is needed to elucidate their mechanisms of action and assess their efficacy in clinical settings. Therefore, generally and from the literature, phenolics including flavonoids play very important roles in diseases because of their antioxidant, anti-inflammatory, and cytotoxic activities [64,65,66].

Based on the docking results presented in Table 3, it is evident that these phenolic compounds exhibit significant interactions with the active site of COX-2, underscoring their potential as lead candidates for further investigation and development as anti-inflammatory agents.

The crystallized ligand (S58), which served as our reference compound, demonstrated the highest affinity for the COX-2 binding site, with a binding energy of −10.8 kcal/mol. This suggests a strong interaction between S58 and the active site of the enzyme. Similarly, chrysin, another phenolic compound under investigation, exhibited a notable binding energy of −10.3 kcal/mol, indicating a favorable binding affinity with COX-2. The docking poses of chrysin with COX-2 (PDB: 1CX2), as illustrated in Figure 1, depicted the orientation of the ligand within the active site, highlighting the specific interactions responsible for its binding. Furthermore, compounds such as hesperetin, baicalein, morin, and catechin also displayed substantial binding energies ranging from −9.4 to −9.8 kcal/mol. These findings suggest that these phenolic compounds have a propensity to interact effectively with the COX-2 enzyme, potentially exerting inhibitory effects on its activity.

The binding affinities observed between S58 and flavonoids such as chrysin with COX-2 are notable; however, their binding patterns and molecular interactions vary significantly due to their distinct chemical compositions. S58, consisting of sulfonamide, trifluoromethyl, and phenyl moieties, establishes specific interactions with the active site residues of the enzyme. Notably, the trifluoromethyl and N2 of the pyrazole moiety form critical hydrogen bonds with amino acid residues (Arg120 and Tyr355, respectively) within the active site, contributing to the stabilization of the binding complex. Additionally, the sulfonamide group enhances the ligand’s overall polarity and engages in electrostatic interactions with charged residues of the enzyme (His90). Moreover, the phenyl moiety facilitates hydrophobic interactions with nonpolar residues, effectively anchoring the ligand within the hydrophobic pocket of the active site. These diverse functional groups collectively enable S58 to establish a robust and selective binding mode with the enzyme, thereby contributing to its inhibitory activity.

In contrast, flavonoids like chrysin possess different functional groups such as hydroxyl and phenolic groups, resulting in distinct binding modes and molecular interactions. The presence of hydroxyl groups in chrysin facilitates hydrogen bonding interactions with the enzyme’s active site residues, influencing its binding pattern. Consequently, while the binding energies of S58 and chrysin may appear similar, their binding modes and molecular interactions are fundamentally different. This underscores the necessity of considering these factors beyond just the energy level when comparing their inhibitory activities.

Our chemical analysis sheds light on the structural characteristics and functional groups of chrysin, hesperetin, baicalein, morin, and catechin, elucidating their potential interactions with COX-2 as revealed by molecular docking. Specifically, the presence or absence of specific hydroxyl groups and variations in aromatic ring configurations play a pivotal role in dictating the observed binding affinities and interaction patterns. All of these compounds belong to the flavonoid class of polyphenols and share common structural features including a flavone or flavanol backbone with multiple hydroxyl groups attached to aromatic rings.

The pharmacophore representations in Figure 2 further elucidate these interactions, providing visual insights into the specific molecular features contributing to the binding modes of each compound and highlighting the structure–activity relationships.

Chrysin, characterized by hydroxyl groups on ring A at position 5, facilitates hydrogen bonding with the polar residue Ser353 in the COX-2 binding pocket. These hydrogen bonds stabilize the ligand–protein complex, enhancing chrysin’s binding affinity. Additionally, chrysin forms interactions with hydrophobic residues such as Leu352, Val523, Trp387, Gly526, Ala527, and Val349. It also engages in Pi-cation and Pi-sulfur interactions with Arg120 and Met522. This unique arrangement confers chrysin’s distinct binding mode and contributes to its favorable binding energy.

Hesperetin, with multiple hydroxyl groups on both the A and B rings, exhibits enhanced structural flexibility, enabling it to adopt diverse conformations within the COX-2 active site. The presence of hydroxyl groups at position 5 on the A ring, along with the oxygen on the C ring, facilitates hydrogen bonding with polar residues such as Tyr355 and Ser353, thereby further stabilizing the ligand–protein complex. Additionally, hesperetin engages in crucial hydrophobic interactions including Pi-sigma, Pi-alkyl, alkyl, and amide-Pi stacked interactions between ring B and residues like Ala527, Leu352, and Gly526. Similarly, interactions occur between ring A and residues such as Val116, Leu359, Leu531, and Val349. Furthermore, the methoxy group interacts with residues like Tyr385, Leu384, and Trp387. These collective interactions contribute significantly to enhancing hesperetin’s binding affinity and overall anti-inflammatory potential.

Baicalein, distinguished by an additional hydroxyl group at position 6 on the A ring, engages in distinctive interactions with COX-2 residues due to its altered steric hindrance and electronic properties. The hydroxyl group at position 5 forms hydrogen bonds with nearby residues, particularly Ser353, thereby influencing baicalein’s orientation and conformation within the binding pocket. Moreover, baicalein exhibits significant hydrophobic interactions between its aromatic rings and residues such as Val523, Trp387, Gly526, Ala527, and Val349. Additionally, baicalein demonstrates Pi-sigma interactions with Arg120 and Pi-sulfur interactions with Met522, further contributing to its stability within the COX-2 active site and enhancing its anti-inflammatory efficacy.

Morin, similar to chrysin, it lacks a hydroxyl group at position 3 on the A ring, which imparts a specific orientation within the COX-2 binding pocket. However, it possesses a hydroxyl group at position 2′ on the B ring, facilitating hydrogen bonding interactions with polar residues like Ser353. This interaction may contribute to its overall anti-inflammatory activity by modulating its interactions with COX-2. Additionally, morin engages in hydrophobic interactions with residues such as Leu352 and Val523, which serve to stabilize the ligand–protein complex. Furthermore, a Pi-cation interaction between the B ring and Arg120 further enhance the binding affinity of morin within the COX-2 active site.

Catechin forms hydrogen bonds with residues like Ser530, utilizing the hydroxyl group at position 3 of the dihydropyran heterocycle (C-ring), thereby exhibiting versatile interactions with COX-2 residues. Additionally, catechin engages in hydrophobic interactions with residues such as Ala527 and Gly526. The strategic positioning of these hydroxyl groups enables catechin to adopt stable conformations within the binding site, thereby enhancing its adaptability and binding affinity to COX-2. Furthermore, the aromatic ring structures present in catechin facilitate π–π interactions with aromatic residues in the COX-2 active site, which further stabilizes the ligand–protein complex and augments its anti-inflammatory potential.

These structural nuances have profound implications for the pharmacological activity and bioavailability of these compounds as variations in functional groups and aromatic ring configurations can modulate their interactions with biomolecular targets. By elucidating the intricate structure–activity relationships of these phenolic compounds, our study aims to provide valuable insights into their therapeutic potential as anti-inflammatory agents.

Upon closer examination, the binding sites and interaction patterns of the crystallized ligand S58 and the flavonoid derivatives (chrysin, hesperetin, baicalein, morin, and catechin) with COX-2 were notably distinct due to their unique structural features. The crystallized ligand S58, characterized by its sulfonamide, trifluoromethyl, and phenyl groups, forms specific interactions with key residues such as Arg120 and Tyr355 through hydrogen bonds and hydrophobic contacts, contributing to its high binding affinity. In contrast, the flavonoid chrysin, with its hydroxyl groups on the A ring, primarily interacts with Ser353 via hydrogen bonding and engages in hydrophobic interactions with residues like Leu352 and Val523.

The additional hydroxyl group on baicalein at position 6 enhances its interaction with Ser353 and modifies its hydrophobic interactions, leading to a distinct binding conformation compared to chrysin. Morin, which lacks a hydroxyl group at position 3 on the A ring but has an additional hydroxyl group on the B ring, forms unique hydrogen bonds with Ser353 and exhibits different hydrophobic contacts, contributing to its specific binding mode within the COX-2 active site. Catechin, with its dihydropyran heterocycle, demonstrates versatile interactions including significant hydrogen bonding with Ser530 and hydrophobic interactions with Ala527 and Gly526, which stabilize its binding conformation and enhance its binding affinity.

These interactions are illustrated in Figure 1, which depicts the 2D interaction modes of S58 and the best-docked phytocompounds with COX-2. This figure provides a visual representation of the specific molecular interactions, highlighting the distinct binding patterns and the involvement of various amino acid residues within the active site of COX-2.

The molecular dynamic (MD) simulations presented in this study aimed to compare the behavior of chrysin, which exhibited the most promising result in the molecular docking analyses compared to other phenolic compounds. Chrysin’s interaction with the target enzyme, cyclooxygenase-2 (COX-2), was compared to that of the reference molecule S58, the ligand co-crystallized with COX-2. The MD simulations aimed to elucidate the dynamic behavior and energetics of the enzyme–ligand complexes, shedding light on their potential as inhibitors of COX-2 activity. Through the analysis of various parameters including the root mean square deviation (RMSD), root mean square fluctuation (RMSF), radius of gyration (Rg), solvent-accessible surface area (SASA), hydrogen bonding, and energy contributions, insights into the stability and binding characteristics of the complexes were gained.

The RMSD values, indicative of the average deviation from the initial structure during the simulation, showed comparable results between the S58 reference complex and the chrysin complex, suggesting stable interactions in both systems. Similarly, RMSF values, representing the flexibility of the residues, remained consistent across both complexes, indicating minimal fluctuations in protein structure.

Analysis of Rg, a measure of protein compactness, revealed no significant differences between the S58 and chrysin complexes, indicating similar overall protein conformations. Furthermore, SASA values, depicting the solvent-exposed surface area, exhibited marginal variations between the two complexes, suggesting comparable degrees of protein exposure to the solvent environment.

The number of hydrogen bonds formed within the complexes showed a slightly higher count in the S58 reference complex compared to the chrysin complex. This discrepancy may reflect the specific interactions favored by each ligand and their binding modes within the active site of the enzyme.

Despite minor variations in hydrogen bonding and energy contributions, the MD simulations underscored the potential of chrysin as a promising inhibitor candidate for COX-2. Its stable interaction with the enzyme and comparable dynamics to the reference molecule highlight its efficacy as a potential therapeutic agent.

Examining the energy contributions, the van der Waals, electrostatic, and polar salvation energies displayed analogous trends between the S58 and chrysin complexes. However, slight differences in the magnitude of these energy terms were observed, likely attributable to variations in the chemical properties of the ligands and their interactions with the protein environment.

Overall, the MD simulations provide valuable insights into the interaction between chrysin and COX-2, emphasizing its potential as a natural inhibitor of inflammatory pathways. Further studies exploring the specific binding mechanisms and therapeutic implications of chrysin are warranted to fully elucidate its pharmacological profile and clinical relevance.

## 5. Conclusions

In summary, our study unveiled the rich phenolic profile of the AAH extract, characterized by diverse compounds including gallic acid, protocatechuic acid, catechin, and notably, chrysin. The extract demonstrated potent antioxidant and anti-inflammatory activities, as evidenced by its high total phenolic content, significant DPPH scavenging capacity, and superior efficacy compared to standard drugs like diclofenac and ketoprofen. Moreover, the molecular docking and dynamic simulations highlight chrysin as a promising COX-2 inhibitor, suggesting its potential as a therapeutic agent for inflammatory disorders. The stable interaction and comparable dynamics of chrysin with the reference ligand underscore its efficacy and merit further investigation. Our findings not only contribute to the understanding of the bioactive composition of the AAH extract, but also shed light on its pharmacological potential for mitigating oxidative stress and inflammation-related conditions. The extract’s lower cytotoxicity compared to potassium dichromate further enhances its appeal as a safer alternative for various applications. Moving forward, targeted studies exploring the molecular mechanisms and clinical implications of these bioactive compounds are essential for unlocking their full therapeutic potential. Harnessing the benefits of the AAH extract may pave the way for the development of novel therapeutic strategies and interventions for inflammatory diseases and other health conditions.

## Figures and Tables

**Table 1:** Total phenolic, flavonoid, and tannin contents along with the antioxidant (DPPH), anti-inflammatory (BSA inhibition), and cytotoxic (brine shrimp lethality bioassay) activities of the AAH extract compared to standards.

	TPC mg GAE/g DM	TFC mg QE/g DM	TTC mg CE/g DM	DPPH IC_50_ µg/mL	Anti-Inflammatory IC_50_ µg/mL	Cytotoxic LC_50_ µg/mL
AAH extract	191 ± 0.03	80.82 ± 0.02	51.91 ± 0.01	19.44 ± 1.1 d	35.73 ± 1.3 a	28.84 ± 2.1 b
BHA	/	/	/	11.94 ± 1.2 b	/	/
BHT	/	/	/	5.5 ± 2.3 a	/	/
Ascorbic acid	/	/	/	15.03 ± 1.6 c	/	/
Alpha tocopherol	/	/	/	14.71 ± 0.9 c	/	/
Ketoprofen	/	/	/	/	164.79 ± 2.3 c	/
Diclofenac	/	/	/	/	63.78 ± 2.1 b	/
BP	/	/	/	/	/	15.73 ± 2.1 a

BHA: butylated hydroxyanisole. BHT: butylated hydroxytoluene. BP: K_2_Cr_2_O_7_ (potassium dichromate). Significant differences exist between the values with various letters (a–d) (*p* < 0.05).

**Table 2:** LC–ESI–MS/MS results of the major phenolic compounds identified in the AAH extract.

No	Name	(M-H)	M2	Retention Time	Phytochemical Class	Ion Mod
1	Gallic acid	169	125	3.218	Phenols	Negative
2	Protocatechuic acid	153	112, 109	5.449	Phenols	Negative
3	Catechin	288	245	6.904	Phenols	Negative
4	Chlorogenic acid	353	191	7.378	Phenols	Negative
5	*o*-Hydroxybenzaldehyde	121	108, 102	7.679	Aldehyde	Negative
6	Vanillic acid	167	153, 122	7.791	Phenols	Negative
7	Syringic acid	197	183, 166	8.401	Phenols	Negative
8	Salicylic acid	137	94, 64, 75	9.539	Phenols	Negative
9	*trans*-Ferulic acid	193	133, 115, 104	10.132	Phenols	Negative
10	Sinapic acid	223	210	10.414	Phenols	Negative
11	Scutellarein-*O*-hexouronide (Scutellarin)	461	369, 286	11.151	Flavone glycoside	Negative
12	*P*-Coumaric acid	163	141	11.502	Phenols	Negative
13	Protocatechuic acid ethyl ester	181	162	11.622	Phenols	Negative
14	Hesperetin-*O*-deoxyhexosyl hexoside (Hesperetin rutinoside)	609	304, 365	11.842	Flavanone glycoside	Negative
15	Quercetin-*O*-deoxyhexosyl hexoside (Rutin)	609	301, 286	12.293	Flavonol glycoside	Negative
16	Quarcetin pentoside	432	315	12.433	Flavonol glycoside	Negative
17	Kaempferol-*O*-hexoside (Kaempferol-*O*-glucoside)	447	284, 255	12.500	Flavonol glycoside	Negative
18	Baicalein-*O*-hexouronide (Baicalin)	445	425	13.287	Flavone glycoside	Negative
19	Morin	300	215	13.327	Flavonol aglycone	Negative
20	Fisetin	285	137, 131	13.900	Flavonol aglycone	Negative
21	Chrysin	253	188	14.230	Flavonone aglycone	Negative
22	Quercetin	301	216	14.821	Flavonol aglycone	Negative
23	Naringenin	271	177	14.999	Flavanone aglycone	Negative
24	Hesperetin	301	286, 242, 199	15.815	Flavanone aglycone	Negative
25	Kaempferol	285	217, 133	16.431	Flavonol aglycone	Negative
26	Baicalein	269	147	17.084	Flavone aglycone	Negative
27	Luteolin	285	155, 161	17.909	Flavone aglycone	Negative
28	Biochanin A	283	121	17.91	Isoflavone aglycone	Negative

**Table 3:** Best docking results of AAH phenolic compounds with cyclooxygenase-2 (COX-2) (PDB:1CX2).

		Binding Energy (Kcal/mol)	Hydrogen Interactions (Distance Å)	Hydrophobic Interactions	Electrostatic Interactions
1	Crystallized ligand (S58)	−10.8	F2-Arg120 (2.32), F3-Arg120 (2.44) F3-Tyr355 (2.58) N2-Tyr355 (3.06)	Val349, Val523, Ser353, Ala527, Leu352, Phe381, Leu384, Trp387, Tyr385	His90
2	Chrysin	−10.3	Ser353 (2.56)	Leu352, Val523, Trp387, Gly526, ala527, Val349	Met522, Arg120
3	Hesperetin	−9.8	O2-Tyr355 (1.86) O5-Tyr355 (2.71)	Tyr385, Leu384, Trp387, Ala527, Gly526, Val116, Leu352, Leu359, Val349, Leu531	-
4	Baicalein	−9.7	Ser353 (2.47)	Val523, Trp387, Gly526, Ala527, Val349	Met522, Arg120
5	Morin	−9.5	Ser353 (3.40)	Val523, Leu352, Gly526, Val349, Ala527, Leu531	Arg120
6	Catechin	−9.4	Met522 (2.29), Ala527 (2.82), Ser530 (3.42)	Gly526, Ala527, Phe518, Leu352, Leu531, Val349	-

**Table 4:** The average values of RMSD, RMSF, Rg, SASA, and the number of H-bonds for the studied complexes.

Complex	Average RMSD (nm)	Average RMSF (nm)	Average Rg (nm)	Average SASA (nm^2^)	H-Bond
1CX2-S58	0.13 ± 0.01	0.07 ± 0.03	2.30 ± 0.06	231.29 ± 3.47	6
1CX2-Chrysin	0.14 ± 0.01	0.07 ± 0.04	2.30 ± 0.07	232.67 ± 2.73	5

**Table 5:** Results showing the van der Waals, electrostatic, polar salvation, SASA, and binding energy in kJ mol^−1^ for the studied complexes.

Protein–Ligand Complex	Van der Waals Energy	Electrostatic Energy	Polar Salvation	SASA Energy	Total Energy (kJ mol^−1^)
1CX2-S58	−151.45 ± 9.03	−15.07 ± 1.22	26.57 ± 7.84	−7.16 ± 0.30	−147.11 ± 11.38
1CX2-Chrysin	−139.11 ± 12.45	−12.66 ± 1.17	17.41 ± 5.71	−4.73 ± 0.25	−139.09 ± 13.21

## Data Availability

Data is contained within the article.
